# Exploitation of Key Regulatory Modules and Genes for High-Salt Adaptation in Schizothoracine by Weighted Gene Co-Expression Network Analysis

**DOI:** 10.3390/ani15010056

**Published:** 2024-12-29

**Authors:** Luo Lei, Xingxing Deng, Fei Liu, He Gao, Yuting Duan, Junting Li, Suxing Fu, Hejiao Li, Yinhua Zhou, Rongrong Liao, Haiping Liu, Chaowei Zhou

**Affiliations:** 1 College of Fisheries, Southwest University, Chongqing 402460, China; leiluo12311@163.com (L.L.); xingxing7965@163.com (X.D.); ddddd926@163.com (Y.D.); llijunting@126.com (J.L.); fsx37999@163.com (S.F.); lhjlhj95szd@163.com (H.L.); fengnian203344@163.com (Y.Z.); lrrong0101@163.com (R.L.); 2 Integrative Science Center of Germplasm Creation in Western China (CHONGQING) Science City, Southwest University, Chongqing 400715, China; fishgaohe@163.com; 3 Livestock and Aquatic Products Affairs Center of Lengshuitan District, Yongzhou 425000, China; 4 Institute of Aquatic Sciences, Tibet Autonomous Region Academy of Agricultural and Animal Husbandry Sciences, Lhasa 851418, China; liufei636@163.com

**Keywords:** *Schizothorax*, high-salt environment adaptation, gills, kidney, liver

## Abstract

Schizothoracine fishes in saltwater lakes of the Tibetan Plateau are important models for studying the evolution and uplift of the Tibetan Plateau. Examining their adaptation to the high-salt environment is interesting. In this study, we first assembled the RNA-Seq data of each tissue of *G. przewalskii*, *G. selincuoensis*, and *G. namensis* from Qinghai Lake, Selincuo Lake, and Namtso Lake, respectively, obtained by the group previously. After obtaining reliable results, the adaptation of the gills, kidneys, and livers of the three species to the high-salinity environment was assessed by weighted gene co-expression network analysis (WGCNA). Using module eigengenes (ME), 21, 22, and 22 gene modules were identified for *G. przewalskii*, *G. selincuoensis*, and *G. nemesis*, respectively. Functional clustering analysis of genes in the significant association module identified several genes associated with osmolarity-regulated potential KEGG pathways in the gills of three species of Schizothoracine fish. Th17 cell differentiation pathway was up-regulated in the gills of all three species; *histocompatibility class 2 II antigen* and *E alpha (h2-ea)* were up-regulated genes in this pathway. Functional clustering analysis of genes in apparently related modules in the kidney unveiled several differential KEGG pathways. The pentose phosphate pathway was up-regulated in the three Schizothoracine fishes, and *glucose-6-phosphate dehydrogenase* (*g6pd*) was an up-regulated gene in this pathway. In the livers of the three *Schizothorax* species, the propanoate metabolism pathway was up-regulated, and *succinate-CoA ligase GDP-forming subunit beta* (*suclg2*) was an up-regulated gene in this pathway. The above analyses provide reference data for the adaptation of *Schizothorax* to high-salt environments and lay the foundation for future studies on the adaptive mechanism of *Schizothorax* in the plateau. These results partly fill the void in the knowledge gap in the survival adaptations of Schizothoracine fishes to highland saline lakes.

## Simple Summary

This study investigates the adaptation of Schizothoracine fishes to high-salinity environments on the Tibetan Plateau, focusing on *G. przewalskii*, *G. selincuoensis*, and *G. namensis* from Qinghai Lake, Selincuo Lake, and Namtso Lake, respectively. RNA-Seq data from the gills, kidneys, and livers of these species were analyzed using weighted gene co-expression network analysis (WGCNA). A total of 21–22 gene modules were identified for each species. Key findings include up-regulation of the Th17 cell differentiation pathway in gills, the pentose phosphate pathway in kidneys, and the propanoate metabolism pathway in livers. Notable up-regulated genes include *h2-ea* in gills, *g6pd* in kidneys, and *suclg2* in livers. These results provide insights into the molecular mechanisms underlying the adaptation of Schizothoracine fishes to saline lakes, offering a foundation for future studies on their survival strategies in highland saline environments.

## 1. Introduction

**Figure 1:**
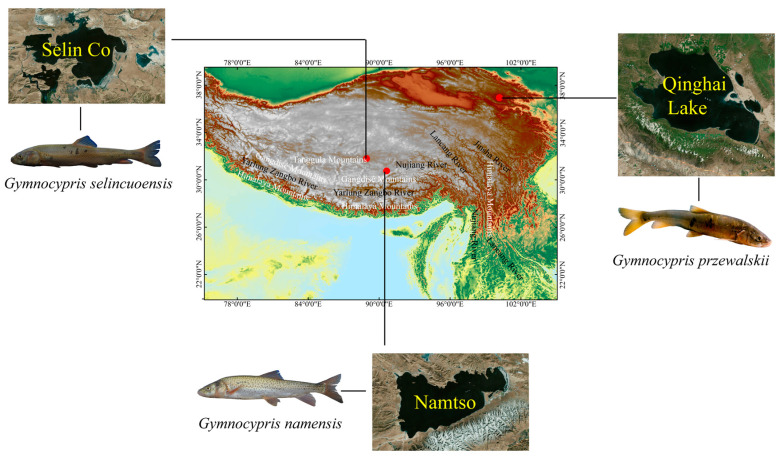
Three saltwater lakes and three species of Schizothoracine fish.

The Qinghai-Tibetan Plateau (QTP), the highest plateau in the world, is home to 1055 lake systems with an area larger than 1 km^2^, making it the world’s largest frozen reservoir after the poles. It is known as the “Water Tower of Asia” [1,2,3]. Approximately 92% of lakes in this region are saline or hypersaline due to the high altitude, dry climate, and extensive exposure to solar radiation [4,5]. Species endemic to lake systems under extreme environmental stresses (high salinity and high altitude) require more energy to support different life processes compared with the traditional lake systems [6], resulting in lower species richness in brackish water lakes than in freshwater lakes [7]. The Tibetan Plateau is a huge laboratory of “evolution in progress.” The Schizothoracine fish in the highland saltwater lakes are important models for studying the evolution and uplift of the Tibetan Plateau [8]. Qinghai Lake, Selin Co, and Namucuo of the Tibetan Plateau are the first-, second-, and third-largest saltwater lakes in China, respectively [9,10,11]. *Gymnocypris przewalskii* (*G. przewalskii*) is the dominant species in Qinghai Lake. *Gymnocypris selincuoensis* (*G. selincuoensis*) and *Gymnocypris namensis* (*G. namensis*) are the only economic fish in Selin Co and Namucuo, respectively (Figure 1). *G. przewalskii* and *G. namensis* are endangered and protected species [12,13,14,15]. *G. przewalskii*, *G. selincuoensis,* and *G. namensis* all belong to the family of nudibranchs (Schizothoracinidae). Owing to the special living environments of the three species and the difficulty in obtaining samples, relevant research is scarce. Existing reports focus on phylogenetics [16], growth and development [17], and resource conservation [18], but few studies have explored their adaptation to the high-salinity environment of saltwater lakes, an interesting question that merits research attention.

Salinity, an important factor in the water environment, affects the physiological function of fish. Environmental salinity affects various physiological mechanisms in fish, such as reproduction, survival, distribution, osmoregulation, and gene expression patterns [19,20]. Fish achieve salinity acclimation mainly by consuming large amounts of energy to synthesize osmotic pressure regulation-related enzymes and proteins [21]. Osmolality regulation in fish is a complex process involving many organs, including the gill, kidney, and liver. The gill is a complex organ composed of cells that perform multiple physiological and metabolic functions. Due to its direct contact with the surrounding environment, the gill has a critical role in gas exchange between water and blood and in osmoregulation, ion regulation, acid–base balance, and nitrogen waste excretion [22]. Fish mainly exclude excess salt from the body through the gills [23]. The kidneys of the fish are an important organ for regulating osmotic pressure [24]. In high-salinity environments, fish balance osmotic pressure by regulating the amount of water they consume. The kidney is the main organ for water reabsorption [25,26]. Osmoregulation is among the most energy-intensive metabolic activities in fish [27]. Therefore, under hypo- or hyper-tonic conditions, fish require massive amounts of energy to maintain osmotic balance [20]. The liver is a key organ for eliciting energy-related metabolic responses in high-salt environments, such as utilizing carbohydrates for osmoregulation [28]. Currently, academics are studying the adaptation of freshwater and marine fishes to salinity stress through various methods. Salinity stress on Nile tilapia (*Oreochromis niloticus*) revealed that long-term salinity disrupts the hepatic function, intestinal health, and gill antioxidative status in Nile tilapia stressed with hypoxia by RNA-Seq [29]. Liver transcriptome analysis reveals extensive transcriptional plasticity during acclimation to low salinity in *Cynoglossus semilaevis* [30]. Yet, few studies have been conducted on the salinity adaptation of fish in highland brackish lakes.

Weighted Analysis of Network Component Activity (WGCNA) [31] is a computational method used to analyze biological networks by assessing the activity of network components and their interactions. It is typically employed in genomics [32], systems biology [33], and bioinformatics [34] to understand complex biological processes and functions. In this study, *G. przewalskii*, *G. selincuoensis*, and *G. namensis* were used as research subjects to explore the adaptation of Schizothoracine fish to high-salt environments by focusing on gill, kidney, and liver tissues through WGCNA analyses. The transcriptome sequencing results of these three fish species were obtained previously by our group.

## 2. Materials and Methods

### 2.1. Material and Data Sources

The samples of *G. przewalskii*, *G. selincuoensis*, and *G. namensis* were collected from Lake Qinghai, Lake Namtso, and Lake Siling Co, respectively, by our group, and RNA-Seq analysis for different somatic tissues was performed [35]. RNA-Seq data for *G. przewalskii* (liver, spleen, kidney, heart, brain, gill, intestinal, muscle, gonads, blood, skin, air bladder, eye), *G. namensis* (gonads, spleen, skin, brain, eye, intestinal, gill, air bladder, kidney, liver, heart, muscle, blood), and *G. selincuoensis* (eye, brain, heart, spleen, kidney, intestinal, gonads, gill, air bladder, muscle, liver) were extracted from the database (https://doi.org/10.6084/m9.figshare.11475294, accessed on 3 March 2024).

### 2.2. Assembly and Analysis of Sequencing Data

Raw reads were first subjected to quality control using fastp to obtain clean reads and ensure data quality [36]. The base composition and quality distribution of the filtered data were analyzed, and Trinity Trinity-v2.15.1 software was used for read assembly [37]. Subsequently, BUSCO v5.8.2 software [38] was used to roughly evaluate the quality of the assembly results based on N50 and the number of genes. Functional annotation of Unigene was performed using four major databases (Nr, SwissProt, KEGG, and COG).

### 2.3. Construction of the Weighted Gene Co-Expression Network (WGCNA) and Potential Association Pathways and Gene Mining

Co-expression networks were constructed using the WGCNA (v1.47) package in R. Gene expression values were imported to construct co-expression modules after filtering genes. Expression correlation coefficients of the remaining genes were then calculated to search for a suitable soft threshold for building gene networks using a scale-free topology model [39,40]. Module eigengenes were used to calculate the correlation coefficients to identify biologically significant modules. Intramodular connectivity (function soft connectivity) of each gene was calculated, and the top 1 or 5% of genes with the highest connectivity were considered ‘hub genes’. The networks were visualized using Cytoscape_3.3.0. For genes in each module, GO and KEGG pathway enrichment analyses were conducted to analyze the biological functions of the modules. A q-value < 0.05 after correction was used as the threshold.

In this study, the expression patterns of the module genes in each tissue are presented in terms of module eigenvalues, and a heat map of sample expression patterns is drawn. Through the sample expression pattern heat map, we can identify the modules that are significantly associated with a particular sample so that the corresponding modules can be subsequently selected for further study. The module eigenvalue is equivalent to the weighted composite value of all gene expressions in the module. Therefore, the values of module eigenvalues in each sample reflect the combined expression levels of all genes in the module in each sample. Since the gill [22,23], kidney [24,25,26], and liver [28] are important tissue organs for regulating osmotic pressure in fish in a high-salt environment, the present study, after identifying the highest expression modules in the gill, kidney, and liver of each species by WGCNA, further screened for differential pathways shared in each tissue organ in the three species, as well as differentially expressed genes common to the pathways. These pathways and genes were then further analyzed to speculate on the possible regulatory mechanisms in response to the high-salt environment.

### 2.4. Structural Analysis

NCBI GenBank is one of the most widely used public nucleic acid sequence databases in the world [41], and all amino acid sequences in this study were obtained from it. DANMAN 6.0 is a data analysis and management software widely used in the field of bioinformatics, usually used for processing DNA sequence data and related bioanalyses [42,43]; it was used for amino acid analysis and multiple sequence comparison in this study. SWISS-MODEL 4.0 is a widely used online protein 3D structure modeling tool based on the principle of homology modeling (homology modeling), which predicts the 3D structure of target proteins from known protein structure templates; the protein 3D model was constructed using the SWISS-MODEL server in this research [44]. MEGA7 v7.0.26 is a reliable bioinformatics analysis software for constructing evolutionary trees based on data from DNA, RNA, or protein sequences that is widely used in biology and evolutionary research [45]. A phylogenetic tree based on protein sequences was constructed using the neighbor-joining (NJ) method in MEGA7 in this research. Evolutionary tree results were considered reliable when Bootstrap values were >70 [46,47,48]. MEME Suite is a widely used and reliable bioinformatics tool suite for discovering and analyzing conserved motifs in sequences; all amino acid sequences were submitted to the MEME Suite database for predicting conserved motifs, and the motif numbers were searched until they exceeded the default thresholds [49]. The NCBI Conserved Domain tool was used to screen the conserved domains of these key genes using default parameters [50].

## 3. Results

### 3.1. De Novo Transcriptome Data and Assembly

**Figure 2:**
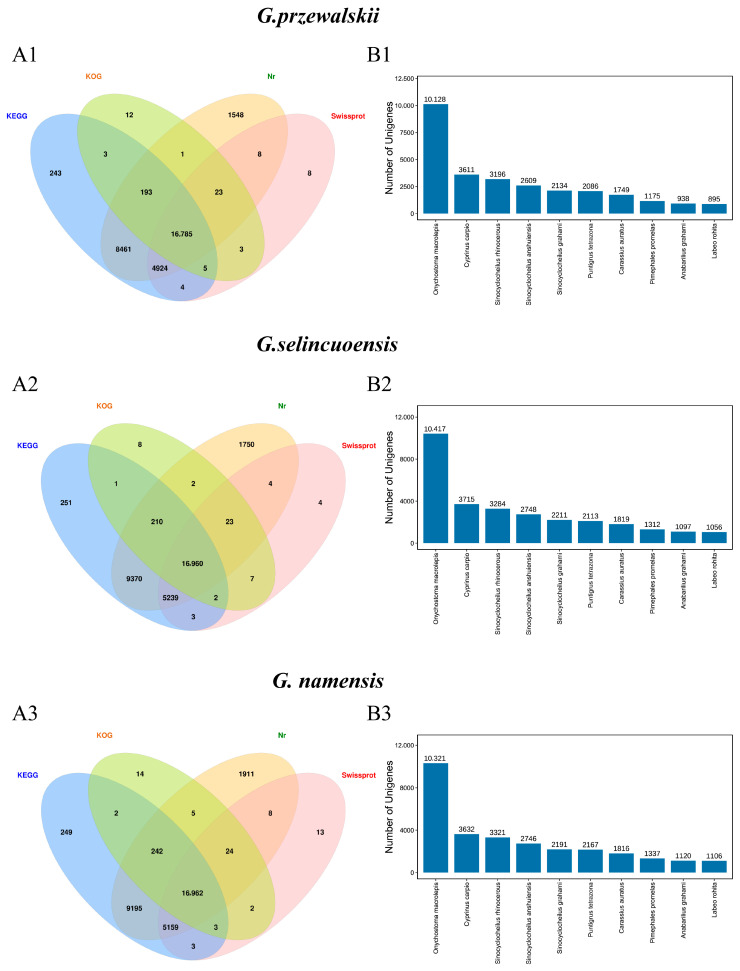
(**A1**–**A3**) Annotated Venn diagrams of the transcriptome sequencing data of *G. przewalskii*, *G. selincuoensis,* and *G. namensis*, respectively. (**B1**–**B3**) Statistical information on the number of comparative species for *G. przewalskii*, *G. selincuoensis*, and *G. namensis*, respectively.

After filtering and screening the raw transcriptome data of the three species of loach fish to obtain high-quality sequences, each library of *G. przewalskii* had clean data of 5.23 Gb with a Q30 base percentage greater than 91.30% (Tables S1 and S2); each library of *G. selincuoensis* had clean data of 5.46 Gb with a Q30 base percentage greater than 91.4% (Tables S3 and S4); and each library of *G. namensis* had clean data of 5.10 Gb with a Q30 base percentage greater than 94.50% (Tables S5 and S6). These results indicate that the quality of the sample sequencing data and de novo assembly was very high, and subsequent experiments utilized these high-quality sequences for analysis. The transcripts of the three species of *Schizothorax* were compared across four major databases (NR, Swiss-Prot, GO, and KEGG) to obtain annotation information, and the statistical results are shown in Figure 2(A1–A3) and Table S7. Additionally, for top-hit species matched against the Nr database, all three species of *Schizothorax* had the highest number of homologous sequence matches with *Onychostoma macrolepis* (Figure 2(B1–B3), Tables S8–S10).

### 3.2. WGCNA and Identification of Target Modules

**Figure 3:**
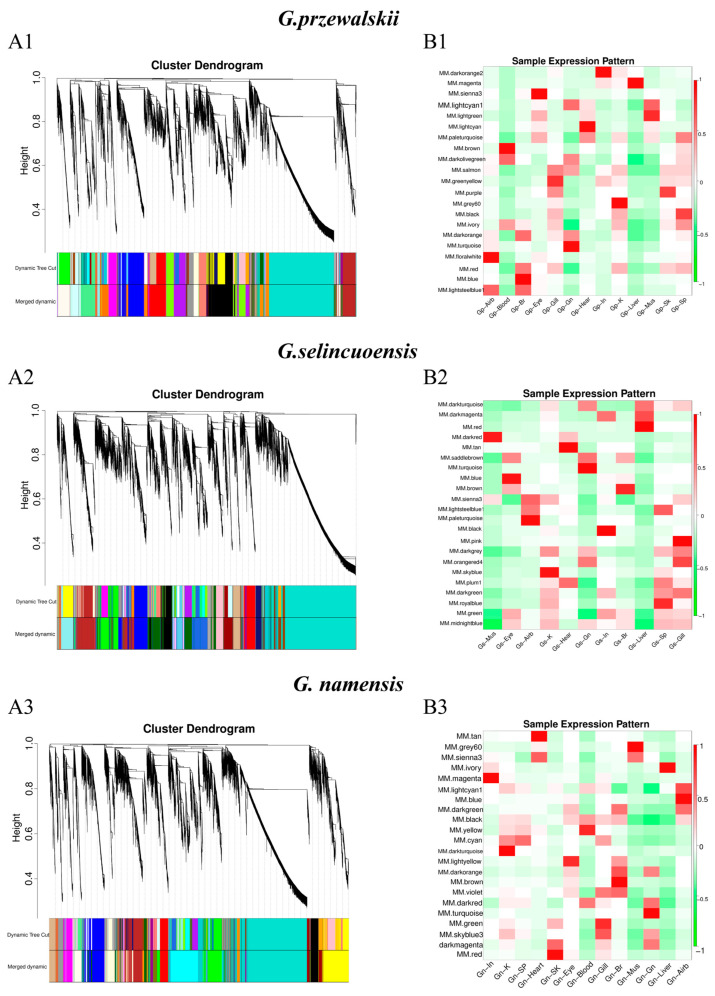
(**A1**–**A3**) Clustering dendrograms for genes of *G. przewalskii*, *G. selincuoensis,* and *G. namensis*, respectively. Dissimilarity was based on topological overlap, together with assigned module colors. Different co-expression modules are shown in different colors. (**B1**–**B3**) Module–trait associations of *G. przewalskii*, *G. selincuoensis,* and *G. namensis*, respectively. Each row corresponds to a module eigengene; each column corresponds to a trait. Red and green represent high and low expression, respectively.

We performed WGCNA to identify genes related to different tissues. Module eigengenes (ME) represented the expression level of genes in each module. ME is the first principal component of a given module and is considered representative of the gene expression profile in the module. The minimum number of genes in each module was set to 50, and a threshold of 0.7 was used to merge similar modules. Finally, 21, 22, and 22 gene modules were identified for *G. przewalskii*, *G. selincuoensis*, and *G. namensis*, respectively (Figure 3(A1–A3)). Modules with common expression patterns were subjected to interaction analysis to identify those significantly associated with different tissues. In *G. przewalskii*, the modules most prominently associated with the gills, kidneys, and liver were the green-yellow, grey60, and magenta modules, respectively (Figure 3(B1)). In *G. selincuoensis*, the modules most prominently associated with the gills, kidneys, and liver were the pink, sky blue, and red modules, respectively (Figure 3(B2)). In *G. namensis*, the modules most prominently associated with the gills, kidneys, and liver were the green, dark turquoise, and ivory modules, respectively (Figure 3(B3)).

### 3.3. Functional Enrichment Analysis for the Target Modules

GO and KEGG enrichment analyses were performed for genes in the modules significant for the gills, kidneys, and liver to explore the adaptation of the three Schizothoracine fish species to high-salt environments. For *G. przewalskii*, GO analysis showed that genes in the green-yellow module corresponding to the gills were mainly enriched in “proteasome core complex”, “spermatoproteasome complex”, and “MHC protein complex” (Figure S1(A1), Table S11). Genes in the grey60 module corresponding to the kidney were mainly enriched in the “secretory granule”, “intrinsic component of the plasma membrane”, and “secretory vesicle” (Figure S1(A2), Table S12). Genes in the magenta module corresponding to the liver were mainly enriched in the “extracellular space”, “extracellular region”, and “cytoplasm” (Figure S1(A3), Table S13). For *G. selincuoensis,* GO analysis showed that genes in the pink module corresponding to the gills were mainly enriched in the “anchoring junction”, “cell-cell junction”, and “tight junction” (Figure S2(A1), Table S14). The genes in the sky blue module corresponding to the kidney were mainly enriched in the “secretory granule lumen”, “intrinsic component of the cytoplasmic side of the plasma membrane”, and “cytoplasmic vesicle lumen” (Figure S2(A2), Table S15). Genes in the red module corresponding to the liver are mainly enriched in the “endoplasmic reticulum”, “endoplasmic reticulum subcompartment”, and “endoplasmic reticulum membrane” (Figure S2(A3), Table S16). For *G. namensis,* GO analysis showed that the genes in the green module corresponding to the gills were mainly enriched in the “cytoplasmic vesicle”, “intracellular vesicle”, and “Vesicle” (Figure S3(A1), Table S17). Genes in the dark turquoise module corresponding to the kidney were mainly enriched in the “intrinsic component of the plasma membrane”, “integral component of the plasma membrane”, and “tertiary granule” (Figure S3(A2), Table S18). Genes in the ivory module corresponding to the liver were mainly enriched in the “peroxisome”, “microbody”, and “cytoplasm” (Figure S3(A3), Table S19).

For *G. przewalskii,* KEGG analysis showed that the genes in the green-yellow module corresponding to the gills were mainly enriched in “Antigen processing and presentation”, “Epstein-Barr virus infection”, and “Pathogenic Escherichia coli infection” (Figure S1(B1), Table S20). Genes in the grey60 module corresponding to the kidney were mainly enriched in the “Metabolic pathways”, “Acute myeloid leukemia”, and “Transcriptional misregulation in cancer” (Figure S1(B2), Table S21). Genes in the magenta module corresponding to the liver were mainly enriched in the “Metabolic pathways”, “Complement and coagulation cascades”, and “Peroxisome” (Figure S1(B3), Table S22). For *G. selincuoensis,* KEGG analysis showed that the genes in the pink module corresponding to the gills were mainly enriched in the “Pathogenic Escherichia coli infection”, “Tight junction”, and “Leukocyte transendothelial migration” (Figure S2(B1), Table S23). Genes in the sky blue module corresponding to the kidney were mainly enriched in “Aldosterone-regulated sodium reabsorption”, “Metabolic pathways”, and “Bile secretion” (Figure S2(B2), Table S24). Genes in the red module corresponding to the liver were mainly enriched in the “Protein processing in the endoplasmic reticulum”, “Metabolic pathways”, and “Glycine, serine and threonine metabolism” (Figure S2(B3), Table S25). For *G. namensis,* KEGG analysis showed that the genes in the green module corresponding to the gills were mainly enriched in “Pathways in cancer”, “Th17 cell differentiation”, and “Endocytosis” (Figure S3(B1), Table S26). Genes in the dark turquoise module corresponding to the kidney were mainly enriched in “Glutathione metabolism”, “Salivary secretion”, and “Bile secretion” (Figure S3(B2), Table S27). Genes in the ivory module corresponding to the liver were mainly enriched in the “Metabolic pathways”, “Peroxisome”, and “Propanoate metabolism” (Figure S3(B3), Table S28).

### 3.4. Potential Regulatory Pathways Underlying High-Salt Adaptation

#### 3.4.1. Critical Osmolarity-Regulating Pathways Related to the Gills of Three Schizothoracine Fish

**Figure 4:**
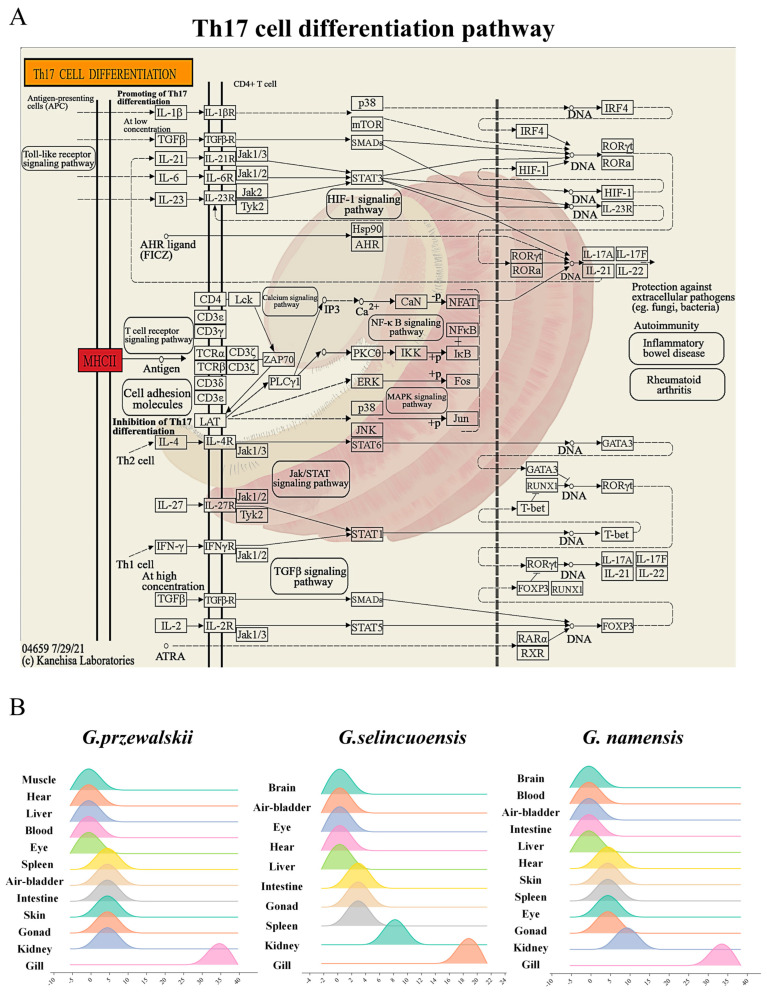
Important pathways and genes regulating osmotic pressure in gills. (**A**) Differential pathways are shared across the three Schizothoracine fish, in particular, the Th17 cell differentiation KEGG pathway. (**B**) Relative expression of *h2-ea* in different tissues of the three Schizothoracine fish.

**Figure 5:**
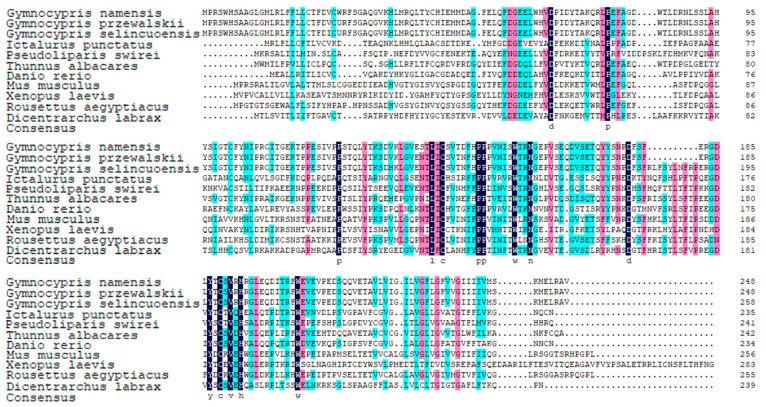
Alignment of the amino acid sequences. GenBank accession numbers: *Mus musculus* (NP_034508.2), *Rousettus aegyptiacus* (XP_036091053.1), *Xenopus laevis* (NP_001090247.1), *Pseudoliparis swirei* (XP_056300596.1), *Thunnus albacares* (XP_044215797.1), *Dicentrarchus labrax* (XP_051269782.1), *Danio rerio* (XP_002660435.3), and *Ictalurus punctatus* (XP_053536026.1). Black shading indicates the same amino acid sequence. Pink shading indicates conserved sequences with more than 75% of the listed polypeptides. The blue shading indicates the conserved sequence with more than half of the listed peptides.

KEGG enrichment analysis of genes expressed in the gills of the three Schizothoracine species revealed that the Th17 cell differentiation pathway was a common differential metabolic pathway, and H2-Ea was significantly up-regulated in the pathway of three Schizothoracinaes (Figure 4A). The relative expression of *h2-ea* was highest in the gill tissues of three Schizothoracine fish (Figure 4B). A comparison of the H2-Ea protein sequences of the three Schizothoracine species revealed that the identity between the three species was very high—all above 93%. The similarity of the H2-Ea protein sequences of the three Schizothoracine fish species with mammals (*Mus musculus*, *Rousettus aegyptiacus*) was over 57%, and with the amphibian (*Xenopus laevis*) H2-Ea protein sequences, it was over 23%; with other freshwater fishes (*Ictalurus punctatus*, *Danio rerio*), it was over 47%, and with marine fish (*Pseudoliparis swirei*, *Thunnus albacares*, *Dicentrarchus labrax*), the similarity was over 45% (Figure 5).

**Figure 6:**
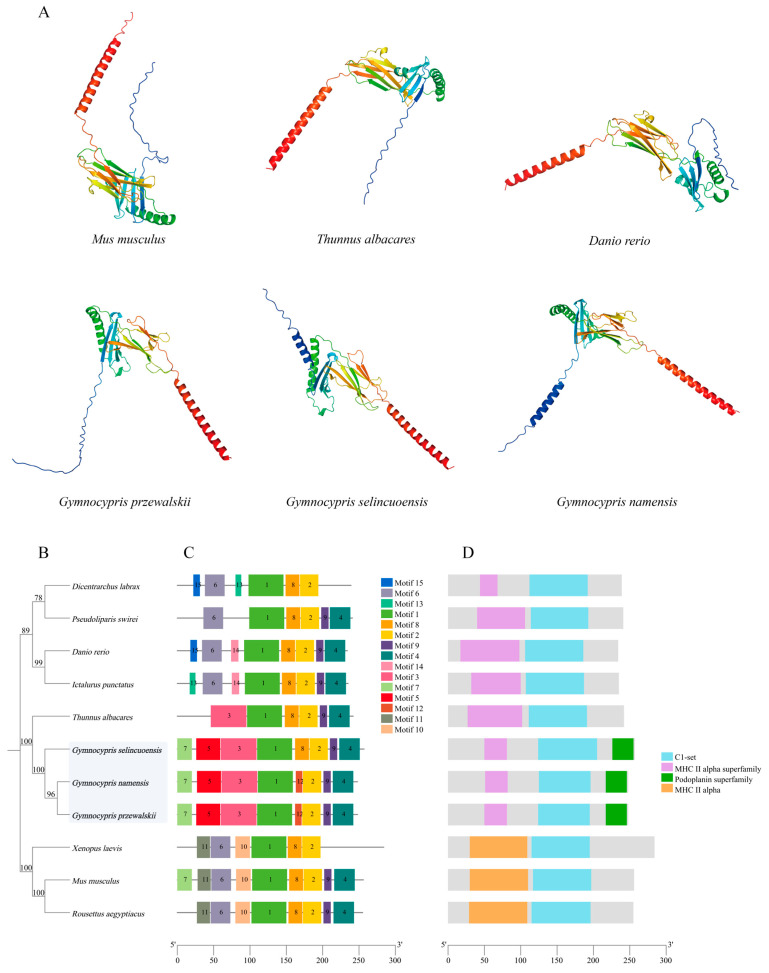
(**A**) Three-dimensional modeling of H2-Ea sequences in *Mus musculus*, *Thunnus albacares*, *Danio rerio*, and the three Schizothoracine fish. (**B**–**D**) Evolutionary, conserved motif, and structural domain analyses of H2-Ea across different species. (**B**) Phylogenetic tree constructed using the NJ method. The bootstrap value is set to 1000. (**C**) Conserved motifs of H2-Ea proteins. Different motifs are represented by colored boxes and different numbers. (**D**) Conserved structural domains of H2-Ea in different species. Sky blue, purple, green, and orange boxes indicate the conserved domain of H2-Ea.

Alpha Fold v2.2.0 was used to predict the three-dimensional structure of H2-Ea sequences from *Mus musculus*, *Thunnus albacares*, *Danio rerio*, and these three species of Schizothoracine fish. The three-dimensional structures of H2-Ea from these six species were very similar, with a core of three β-folded fragments and the peripheral wrapping of four-segmented *α*-helices (Figure 6A). A phylogenetic tree of H2-Ea was constructed using the NJ method with MEGA 11v.11.0. 13 software, and three Schizothoracine H2-Ea sequences were clustered into a single branch in which all bootstrap values were greater than 70. (Figure 6B). Conserved motifs of H2-Ea proteins of 10 species were characterized, and 15 conserved protein motifs were extracted, among which motif 5 was unique in H2-Ea of these three species of Schizothoracine (Figure 6C). All species of H2-Ea proteins have a C1-Set conserved protein structural domain, with *Xenopus laevis*, *Mus musculus*, and *Rousettus aegyptiacus* sharing the MHC Il alpha structural domain and the other eight species sharing the MHC Il alpha superfamily structural domain. Interestingly, the Podoplanin superfamily domain is unique to three species of Schizothoracine fish (Figure 6D).

#### 3.4.2. Critical Pathways Involved in Osmotic Pressure Regulation in the Kidneys of Three Schizothoracine Fish

**Figure 7:**
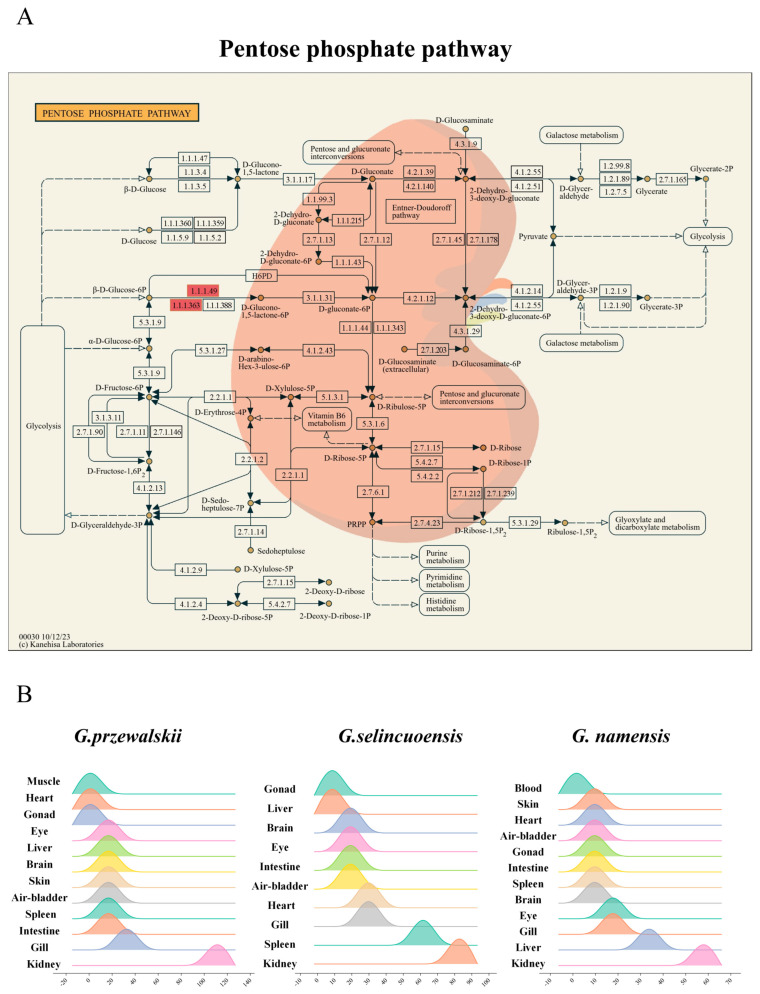
Important pathways and genes that regulate the osmotic pressure in the kidney. (**A**) Differential pathways are shared across three Schizothoracine fish, in particular, the pentose phosphate pathway. (**B**) Relative expression of *g6pd* in different tissues of the three Schizothoracine fish.

**Figure 8:**
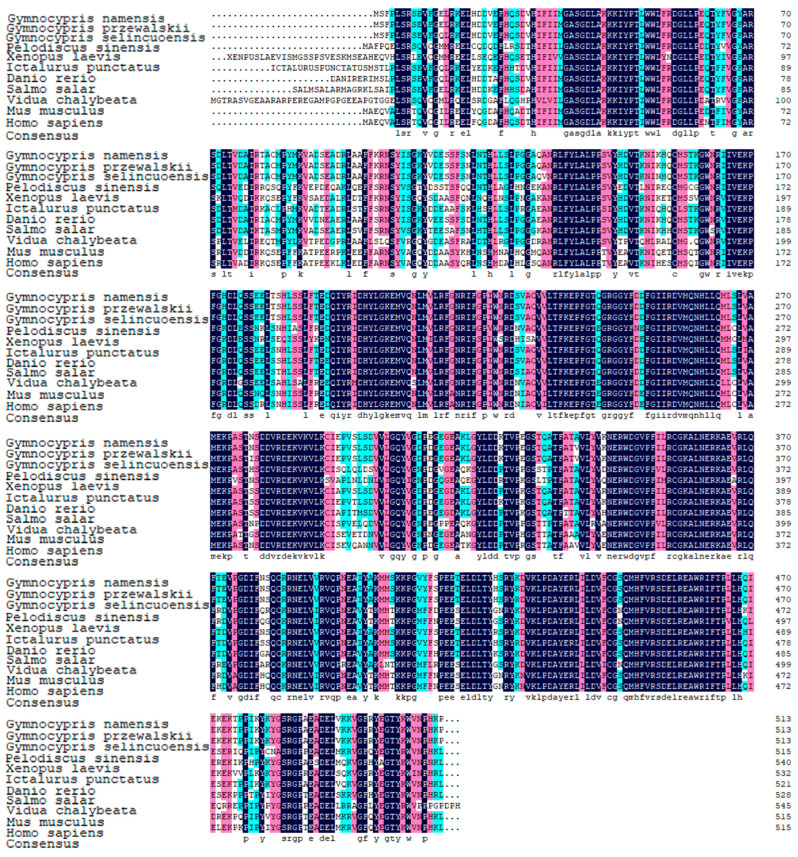
Alignment of amino acid sequences. The structural domain is underlined in yellow. GenBank accession numbers: *Homo sapiens* (AAA92653.1), *Mus musculus* (NP_032088.1), *Vidua chalybeata* (XP_053823932.1), *Pelodiscus sinensis* (XP_006130125.2), *Xenopus laevis* (XP_041427836.1), *Danio rerio* (XP_005162067.1), *Salmo salar* (NP_001135196.1), and *Ictalurus punctatus* (XP_017343061.1). Black shading indicates the same amino acid sequence. Pink shading indicates conserved sequences with more than 75% of the listed polypeptides. The blue shading indicates the conserved sequence with more than half of the listed peptides.

KEGG enrichment analysis of genes expressed in the kidneys of the three Schizothoracine species revealed that the pentose phosphate pathway was a common differential KEGG pathway, and G6PD was significantly up-regulated in this pathway of three Schizothoracinaes (Figure 7A). The relative expression of *g6pd* was the highest in the kidney tissues of the three Schizothoracine fish (Figure 7B). A comparison of the G6PD protein sequences of the three Schizothoracine species revealed that the identity between the three species was very high—all above 98%. The similarity of the G6PD protein sequences of the three Schizothoracine fish species with mammals (*Homo sapiens*, *Mus musculus*) was over 88%; with birds (*Vidua chalybeata*), it was over 69%; with amphibious reptiles (*Pelodiscus sinensis*, *Xenopus laevis*), it was over 73%; with other freshwater fishes (*Ictalurus punctatus*, *Danio rerio*), it was over 91%; and with marine fish (*Salmo salar*), the similarity was over 86% (Figure 8).

**Figure 9:**
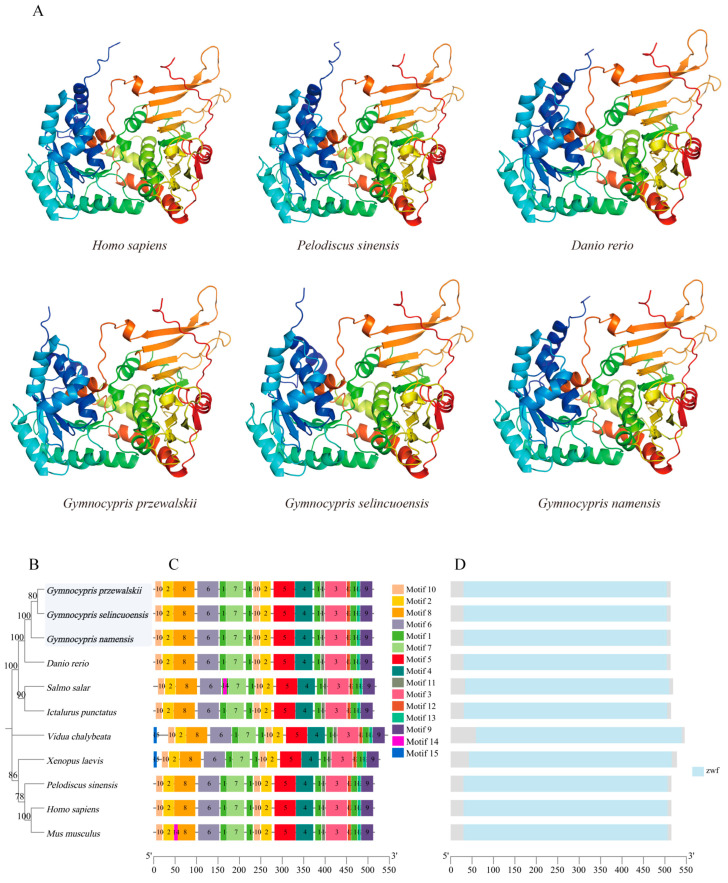
(**A**) Three-dimensional modeling of G6PD sequences of *Homo sapiens*, *Pelodiscus sinensis*, *Danio rerio*, and the three Schizothoracine fish. (**B**–**D**) Evolutionary, conserved motif, and structural domain analyses of G6PD across different species. (**B**) Phylogenetic tree constructed using the NJ method. The bootstrap value is set to 1000. (**C**) Conserved motifs of G6PD proteins. Different motifs are represented by colored boxes and different numbers. (**D**) Conserved structural domains of G6PD in different species. Sky blue boxes indicate the conserved domain of G6PD.

Using Alpha Fold v2.2.0 to predict the 3D structure of the G6PD protein from *Homo sapiens*, *Pelodiscus sinensis*, *Danio rerio*, and these three Schizothoracine fish, a perfect overlap was found, indicating that the G6PD proteins were highly conserved. G6PD proteins have two structural domains. The N-terminal domain comprises three beta-folded segments with multiple alpha-helices, and the C-terminal domain (reddish-orange part) is more loosely structured, mainly comprising beta-folded segments, and the active center is located in the middle of the N- and C-terminal domains (Figure 9A). A phylogenetic tree of G6PD was constructed using the NJ method with MEGA11 software, in which all bootstrap values were greater than 70, and three Schizothoracine G6PD were clustered into a single branch (Figure 9B). The conserved motifs of G6PD proteins of the 11 species were characterized, and 15 conserved protein motifs were found (Figure 9C). All 11 species of G6PD have only one conserved protein structural domain—zwf (Figure 9D).

#### 3.4.3. Critical Pathways Regulating Osmotic Pressure in the Liver of Three Schizothoracine Fish

**Figure 10:**
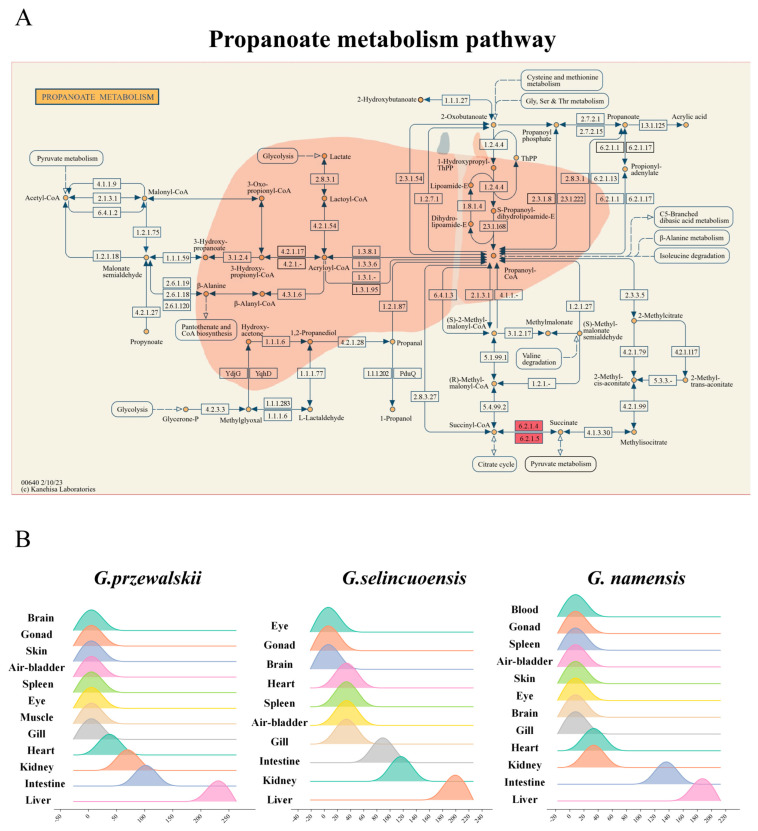
Important pathways and genes regulating osmotic pressure in the liver. (**A**) Differential pathways are shared across the three Schizothoracine fish, in particular, the propanoate metabolism pathway. (**B**) Relative expression of *suclg2* in different tissues of three Schizothoracine fish.

**Figure 11:**
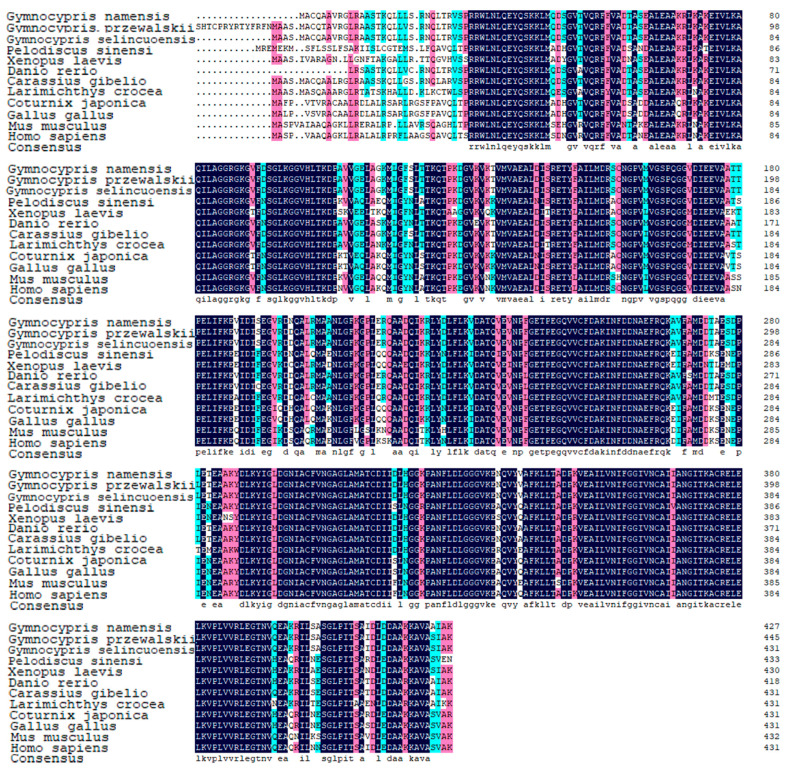
Alignment of the amino acid sequences. The structural domain is underlined in green. GenBank accession numbers: *Homo sapiens* (AAH68602.1), *Mus musculus* (NP_035637.2), *Coturnix japonica* (XP_015730848.1), *Gallus gallus* (NP_001006141.1), *Pelodiscus sinensis* (XP_006120066.2), *Xenopus laevis* (NP_001089908.1), *Carassius gibelio* (XP_052424800.1), *Danio rerio* (NP_001373238.1), and *Larimichthys crocea* (XP_010735540.3). Black shading indicates the same amino acid sequence. Pink shading indicates conserved sequences with more than 75% of the listed polypeptides. The blue shading indicates the conserved sequence with more than half of the listed peptides.

KEGG enrichment analysis of genes expressed in the liver of the three Schizothoracine species revealed that the propanoate metabolism pathway was a common differential KEGG pathway, and SUCLG2 was significantly up-regulated in the pathway of three Schizothoracinaes (Figure 10A). Relative expression of *suclg2* was highest in the liver tissues of the three Schizothoracine fish (Figure 10B). A comparison of the SUCLG2 protein sequences of the three Schizothoracine species revealed that the identity between the three species was very high—all above 95%. The similarity of the SUCLG2 protein sequences of the three Schizothoracine fish species with mammals (*Homo sapiens*, *Mus musculus*) was over 86%; with birds (*Coturnix japonica*, *Gallus gallus*), it was over 90%; with amphibious reptiles (*Pelodiscus sinensis*, *Xenopus laevis*), it was over 83%; with other freshwater fishes (*Carassius gibelio*, *Danio rerio*), it was over 93%; and with marine fish (*Larimichthys crocea*), it was over 86% (Figure 11).

**Figure 12:**
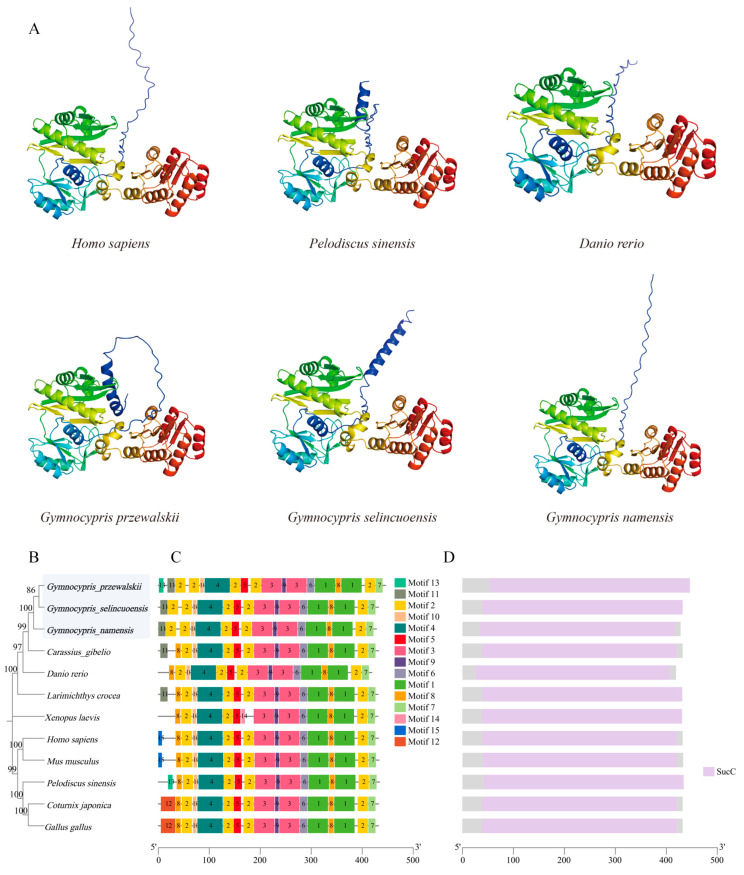
(**A**) Three-dimensional modeling of SUCLG2 sequences of *Homo sapiens*, *Pelodiscus sinensis*, *Danio rerio*, and the three Schizothoracine fish. (**B**–**D**) Evolutionary, conserved motif, and structural domain analyses of SUCLG2 in different species. (**B**) Phylogenetic tree constructed using the NJ method. The bootstrap value is set to 1000. (**C**) Conserved motifs of SUCLG2 proteins. Different motifs are represented by colored boxes and different numbers. (**D**) Conserved structural domains of SUCLG2 in different species. Pink boxes indicate the conserved domains of SUCLG2.

The N-terminal structural domain of the SUCLG2 protein (indicated in green and yellow) is the most complex and diverse part (Figure 12A). This part comprises many different amino acid sequences, forming an intricate three-dimensional structure. Nevertheless, the overall layout of the N-terminal structural domain shows a certain regularity, with the alpha helix (α-helix) constituting its peripheral structure and the beta fold (β-sheet) constituting its core. This unique structural design provides the protein with the necessary stability and the necessary flexibility for its biological function. In contrast to the N-terminal structural domain, the C-terminal structural domain of the SUCLG2 protein (indicated in red and orange, respectively) is relatively simple. Despite the simplified sequence and structure, the C-terminal structural domain follows a similar layout as the N-terminal. A phylogenetic tree of SUCLG2 was constructed using the NJ method with MEGA11 software, in which all bootstrap values were greater than 70, and SUCLG2 sequences of the Schizothoracine fish were clustered into a single branch (Figure 12B). The conserved motifs of SUCLG2 proteins of the 12 species were characterized, and 15 conserved protein motifs were found (Figure 12C). The SUCLG2 sequence of all 12 species showed conserved protein structural domains, that is, SucC (Figure 12D).

## 4. Discussion

The adaptation of high-altitude fish has remained a research focus, and how fish in high-altitude saline lakes adapt to their environment is particularly complex and fascinating. This study focuses on *G. przewalskii*, *G. selincuoensis*, and *G. namensis* from China’s largest saline lakes—Qinghai Lake, Selin Co, and Namucuo. This is the first study to use WCGNA to explore the adaptation of the gills, kidneys, and liver of these three species of fish to high-altitude, high-salinity lakes.

### 4.1. Response of Gills of Three Species of Schizothoracine Fishes to High-Salt Stress

The gill is an important immune organ essential for the adaptation of fish to high-salt environments. It is mainly involved in salt excretion, salt absorption, water regulation, and maintenance of ionic homeostasis to ensure that fish maintain a normal physiological state [22,51,52,53]. On exploring the adaptation of the gill of *Acanthogobius ommaturus* to high-salinity environments, cytokine–cytokine receptor interaction, metabolism of xenobiotics by cytochrome P450, and proximal tubule bicarbonate reclamation were identified as crucial KEGG metabolic pathways [54]. In *Oryzias melastigma* [55], glycolysis/gluconeogenesis, methane metabolism, carbon metabolism, biosynthesis of antibiotics, biosynthesis of amino acids, fructose and mannose metabolism, microbial metabolism in diverse environments, amino sugar and nucleotide sugar metabolism, and cytokine–cytokine receptor interaction were important KEGG metabolic pathways for gill adaptation to high-salinity environments. The proteasome core complex, a pathway commonly associated with fish adaptation to high-salt stress, was also found in the gills of *G. przewalskii* in this study. The proteasome core complex plays multiple functions in the adaptation of fish to high salinity; it helps fish cells maintain normal function by regulating protein degradation and minimizing cell damage from salt stress, thus improving the survival of fish cells in high-salt environments [56,57]. For example, under high-salt conditions, the level of ubiquitination in Thunnus albacares cells is significantly increased and proteasome activity is enhanced, which can regulate proteins related to ion channels, antioxidant enzymes, and other proteins, thus helping fish to adapt to the high-salt environment [58]. However, in this study, we uncovered that the Th17 cell differentiation pathway, rarely reported previously, may be closely related to the gill adaptation of the three species of Schizothoracine to high-salinity environments.

The Th17 cell differentiation pathway was significantly up-regulated in the gills of all three species of Schizothoracine fish. Prolonged exposure to high salinity suppresses the immune system in fish [59,60], and Th17 cells are a special class of T helper cells whose differentiation pathways and functions are important for the immune system [61]. Th17 cells can directly activate the local immune response by secreting cytokines, such as IL-17A and IL-17F, which promote the production of various inflammatory mediators (such as IL-6 and TNF-α), thus increasing the intensity of the inflammatory response against bacterial or fungal infections [62,63]. IL-22 is associated with tissue repair and can promote cell proliferation and tissue regeneration [64]. Th17 cells can help repair damaged tissues by secreting IL-22, especially that of mucosal tissues [65]. Th17 cells can regulate the overall immune response through interactions with other immune cells, such as Th17 cells, which can secrete IL-21 to promote B-cell proliferation and antibody production, thus enhancing the humoral immune response [66,67]. During evolution, species tend to retain those genetic traits that contribute to survival and reproduction through natural selection [68]. The gill serves as the interface between fish and the external environment, and it plays a crucial role in the face of challenges such as high salinity, pathogen invasion, and oxygen demand [69]. In this study, Th17 cells in the gill species of the three species of schizothoracine fish enhance the immune response of the gills by secreting cytokines, such as IL-17, to improve their defense against bacteria or other pathogens, thus promoting the survival of fish in a higher salinity environment, which may be a specific immune strategy of the three species of schizothoracine fish to cope with the high-salinity environment and is a result of their adaptive evolution.

**Figure 13:**
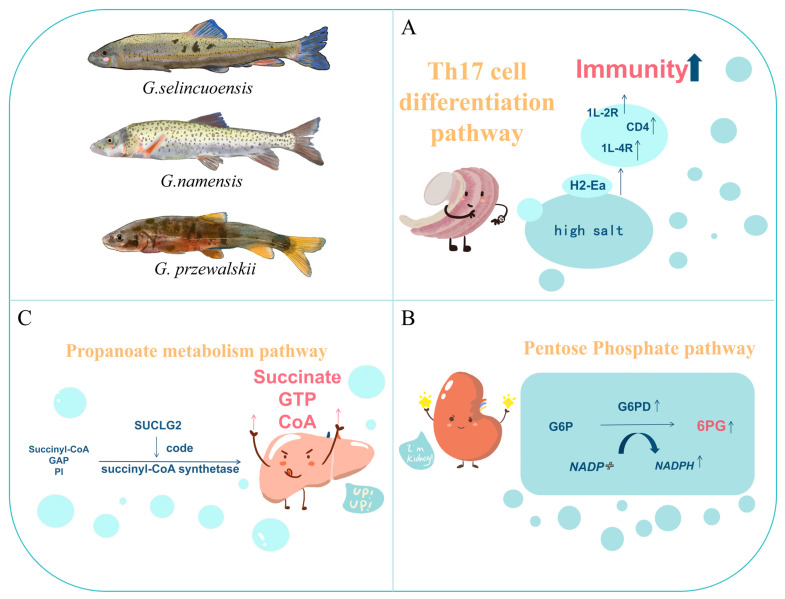
Regulatory pathways in the gills, kidneys, and liver of three species of Schizothoracine fishes potentially respond to high-salt environments. (**A**) H2-Ea in the regulatory pathway involved in the Th17 cell differentiation pathway in gills. (**B**) G6PD regulatory pathways involved in the pentose phosphate pathway in the kidney. (**C**) SUCLG2 regulatory pathways involved in the propanoate metabolism pathway. An upward arrow next to a gene indicates a rise in gene expression.

MHC class II molecules are important antigen-presenting molecules that function primarily in the immune system [70]. H2-Ea is a subtype of MHC II molecules primarily found in certain fish species [71]. H2-Ea was significantly up-regulated in the Th17 cell differentiation pathway in the gill tissues of the three fish. According to the KEGG analysis, H2-Ea was upstream of the Th17 cell differentiation pathway, and significant H2-Ea up-regulation could positively promote a series of immune-related genes downstream, such as 1L-2R [72], 1L-4R [73], and CD4 [74]. Therefore, we speculate that the Schizothoracine fish in the high-salt environment may be subjected to more immune challenges or infections, which may prompt a more active immune system response, leading to the up-regulation of the expression of H2-Ea molecules to enhance the antigen-presenting capacity and immune response (Figure 13A).

### 4.2. Response of the Kidneys of Three Species of Schizothoracine Fish to High-Salt Stress

Salinity tolerance requires a delicate balance between bioenergetic costs and trade-offs that may modulate adaptive plasticity and evolutionary change for kidney function [24]. Undoubtedly, the kidney is an osmotically important organ in fish, and the process of osmotic pressure regulation involves many pathways and genes [75,76,77]. In *Hypophthalmichthys molitrix* [78], the PPAR signaling pathway, metabolism of xenobiotics by cytochrome P450, cytokine–cytokine receptor interaction, glutathione metabolism, and tyrosine metabolism are important KEGG metabolic pathways involved in regulating high-salinity stress in the kidneys. In *Acipenser baeri* [79], glycerophospholipid metabolism, fatty-acid biosynthesis, glycolysis/gluconeogenesis, and oxidative phosphorylation are involved in osmoregulation.

In the present study, the pentose phosphate pathway was found to be a differentially significant KEGG pathway in the kidneys of all three Schizothoracine species. The pentose phosphate pathway crucially regulates the antioxidant status and provides input in energy metabolic pathways [80]. High-salt environments are usually accompanied by increased oxidative stress and excessive production of oxygen-free radicals inside the cells, which may lead to cellular damage [81]. The pentose phosphate pathway helps counteract oxidative stress by producing NADPH, a molecule with a high reducing capacity that provides antioxidant-reducing power to cells [82]. The pentose phosphate pathway produces glucose metabolites in cells that are used for nucleic acid and amino sugar synthesis and are involved in cellular energy metabolism and antioxidant defense [83]. In the present study, the activity of the pentose phosphate pathway was up-regulated in the kidneys of all three species of Schizothoracine, likely to satisfy the additional cellular demand for energy and antioxidant substances. NADPH produced via the pentose phosphate pathway is used for reduction reactions and is important for other physiological processes, such as the synthesis of lipids and the protection of cell membranes from oxidative damage [84].

G6PD is an important enzyme involved in intracellular metabolic processes [85]. G6PD catalyzes the conversion of glucose-6-phosphate (G6P) to 6-phosphogluconate (6PG) and generates NADPH, which functions as a reducing agent and is involved in intracellular reduction reactions, including the synthesis of reduced glutathione [86]. This helps protect cells from oxidative damage during osmotic pressure regulation. NADPH is involved in the synthesis of other important biomolecules, such as the synthesis of lipids and nucleic acids, which are essential for cell membrane function and ion transport [87]. Intriguingly, *g6pd* was a shared, significantly up-regulated gene in the pentose phosphate pathway across all three Schizothoracine species. Kidney cells of all three species of Schizothoracine fish need to process large amounts of ions and water to regulate osmotic pressure, a process prone to oxidative stress. Thus, we speculate that G6PD, significantly up-regulated in all three species of Schizothoracine, can support normal osmotic pressure regulation by promoting NADPH production and maintaining an antioxidant milieu that protects these cells from oxidative damage (Figure 13B). Salinity stress on *M. salmoides* revealed that *M. salmoides* removed toxic substances produced by salinity stress and mitigated oxidative damage by up-regulating UGTs and GSTs, hence maintaining normal physiological function in the kidneys [88]. More interestingly, *Oreochromis niloticus* was subjected to different salinity stresses, and it was found that *g6pd* was suppressed in vivo when *Oreochromis niloticus* was subjected to medium- and high-salinity stresses [89]. The above results indicate that the adaptive mechanisms of the kidneys to high-salt stress vary considerably in different fish species.

### 4.3. Response of the Liver of Three Schizothoracine Fish to High-Salt Stress

The liver contains various metabolic enzymes that help meet increased energy demands in response to environmental stress by facilitating the metabolic breakdown of proteins to produce amino acids (gluconeogenesis) when hepatic glycogen reserves are depleted [90,91]. Changes in salinity in the aquatic environment increase the basal metabolism of fish, in turn increasing their oxygen demand and reactive oxygen species (ROS) production, as well as the osmoregulation of fish during salinity acclimatization, further increasing the energy demand [92]. In *Scophthalmus maximus* [93], key KEGG pathways for liver adaptation to salt stress include glycerophospholipid metabolism, adipocytokine signaling pathway, insulin resistance, PPAR signaling pathway, glycerolipid metabolism, and cytokine–cytokine receptor interaction. Sucrose metabolism, glycerolipid metabolism, galactose metabolism, arachidonic acid metabolism, and the FoxO signaling pathway are important KEGG pathways involved in regulating high-salinity stress in the liver of *Seriola dumerili* [94].

The propanoate metabolism pathway was identified as an important regulatory pathway in the liver of all three Schizothoracine species for coping with high-salinity environments. The propanoate metabolism pathway has several important functions in organisms, including energy metabolism, carbon source utilization, and the synthesis of specific metabolites [95]. Propanoate metabolism contributes to cellular osmotic regulation through multiple mechanisms. It provides energy via pathways, such as propanoate CoA-transferase and methylmalonyl-CoA, which are essential for maintaining cellular functions under varying osmotic conditions [96]. It influences ion gradients across membranes, further impacting osmotic balance. Moreover, propanoate metabolism can regulate the synthesis of osmoprotectants, which is crucial for protecting cells from osmotic stress. These interconnected processes ensure cells maintain optimal turgor pressure and functionality across different osmotic environments. High-salt environments lead to increased osmotic pressure in fish, requiring a series of physiological and metabolic adaptations to maintain water–salt balance in the body [97]. The propanoate metabolism pathway was differentially up-regulated in the livers of the three Schizothoracine species, and we speculate that the livers of the three Schizothoracine species produce more energy by increasing the activity of the propanoate metabolism pathway.

*Suclg2* was significantly up-regulated in the propanoate metabolism pathway in the livers of all three Schizothoracine species. *Suclg2* is mainly expressed in tissues involved in biosynthesis, notably the liver and kidney, and is mainly localized to the mitochondria [98,99]. Succinyl-CoA synthetase is an important enzyme in the tricarboxylic acid cycle (TCA cycle), which is encoded by *suclg2* [100,101,102]. The TCA is an important physiological process regulating the body’s energy production [103,104]. Succinyl-CoA synthetase catalyzes a key step in the TCA, converting succinyl-CoA to succinate and generating GTP. Subsequently, GTP is converted to ATP through various reactions to supply energy requirements for various metabolic activities of the cell [105]. Fish require more energy to regulate osmolality to adapt to high-salt environments [106]. Although there is no direct study on the direct involvement of *suclg2* in osmotic pressure regulation, *suclg2* up-regulation in the livers of the three Schizothoracine species promotes the TCA cycle, which produces more energy and helps them adapt to the high-salt environment (Figure 13C).

## 5. Conclusions

The adaptation of Schizothoracine fish to high-salt water environments involves complex physiological, biochemical, and molecular processes. This study is preliminary research on the adaptation of the gills, kidneys, and liver of *G. przewalskii*, *G. selincuoensis*, and *G. namensis* to high-salt environments. WGCNA revealed that the Th17 cell differentiation KEGG pathway is a common up-regulated pathway in the gills of all three species of Schizothoracine, the pentose phosphate pathway is a common up-regulated pathway in the kidneys, and the propanoate metabolism pathway is a common up-regulated pathway in the liver. These three KEGG pathways have rarely been reported in other studies of high-salt acclimatization in fish. Further analysis revealed that *h2-ea*, *g6pd*, and *suclg2* are important regulators of gill, kidney, and liver adaptation to high-salt environments in three species of Schizothoracine fish, respectively. These results provide new insights into the survival adaptations of Schizothoracine fish in saline lakes. These results not only provide new perspectives on the adaptive mechanisms of Schizothoracine fishes in salt lakes and enrich our understanding of biological adaptation in extreme environments but also allow us to optimize aquaculture management, conserve ecological diversity, and effectively respond to possible future challenges posed by environmental changes and human activities through a deeper understanding of the adaptive mechanisms of fishes.

## Data Availability

Data are contained within the article and supplementary materials.

## Supplementary Materials

The following supporting information can be downloaded at: https://www.mdpi.com/article/10.3390/ani15010056/s1.
