# Supplementary figures and images for: Exploitation of Key Regulatory Modules and Genes for High-Salt Adaptation in Schizothoracine by Weighted Gene Co-Expression Network Analysis
Source: Animals (Basel). 2024 Dec 29;15(1):56. doi: 10.3390/ani15010056 (PMC11718949; doi:10.3390/ani15010056)

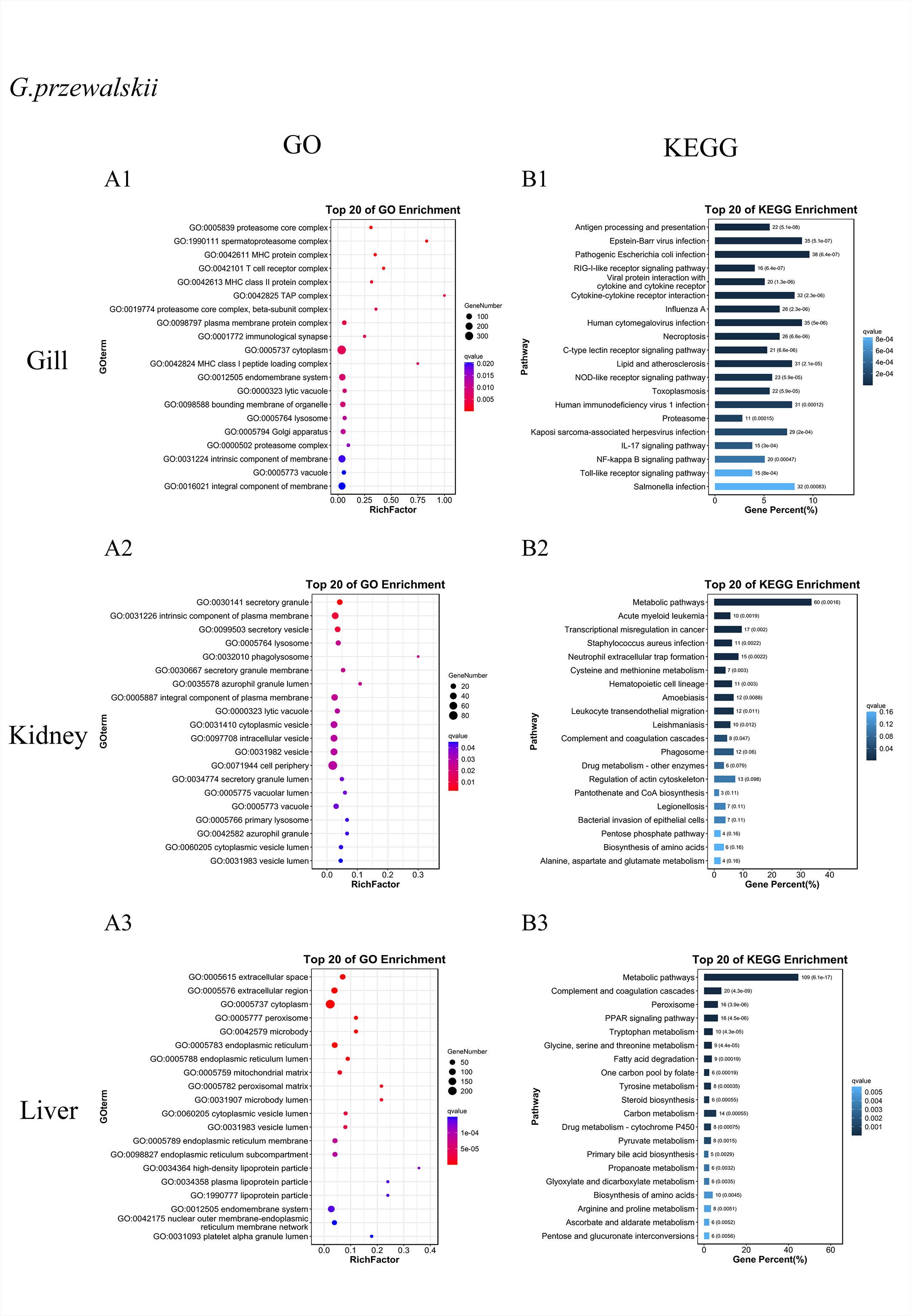

Supplement: Supplementary file 1 [file animals-15-00056-s001.zip › Suppl. Figures and Tables/Figures/Fig S1.tif]

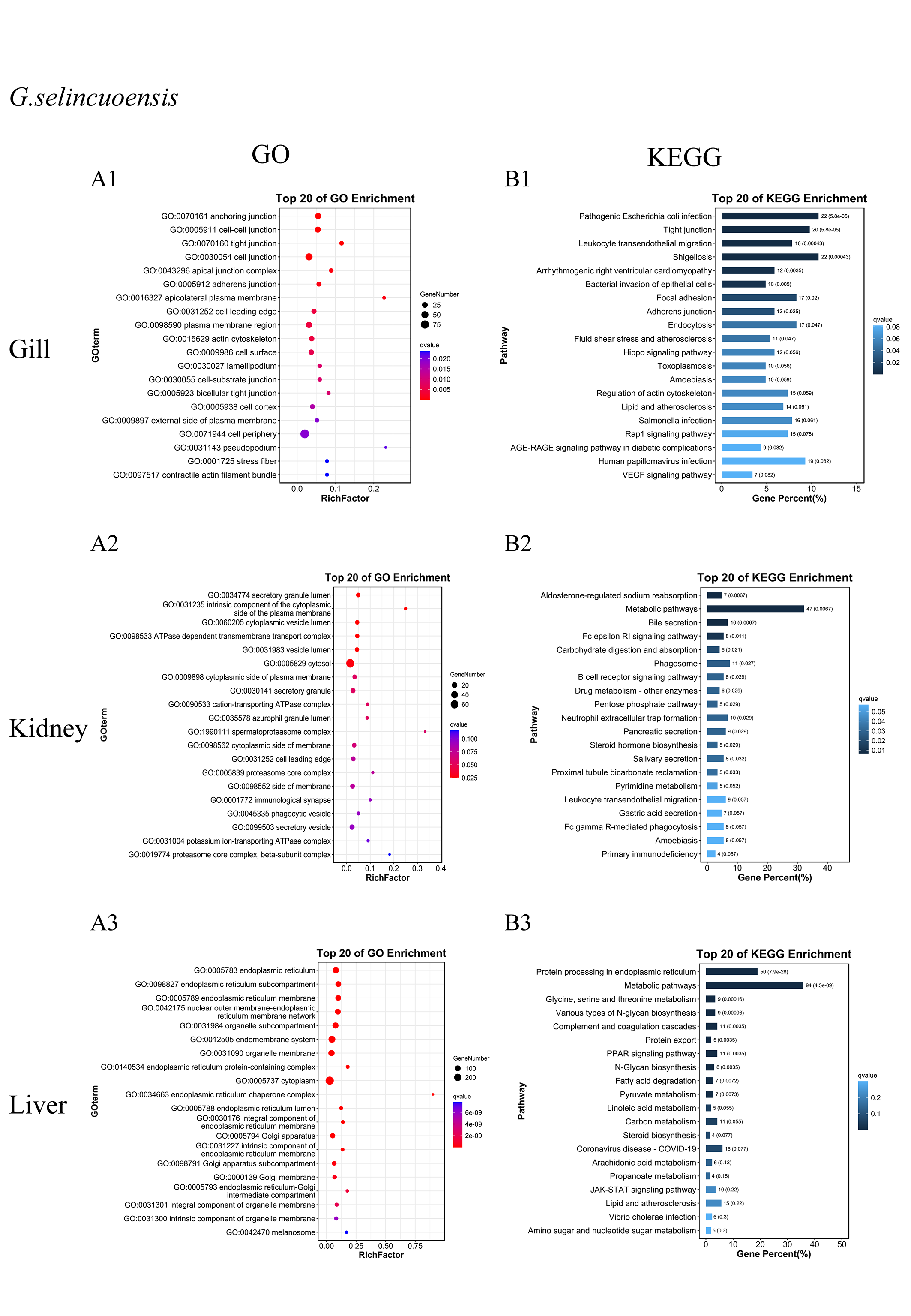

Supplement: Supplementary file 1 [file animals-15-00056-s001.zip › Suppl. Figures and Tables/Figures/Fig S2.tif]

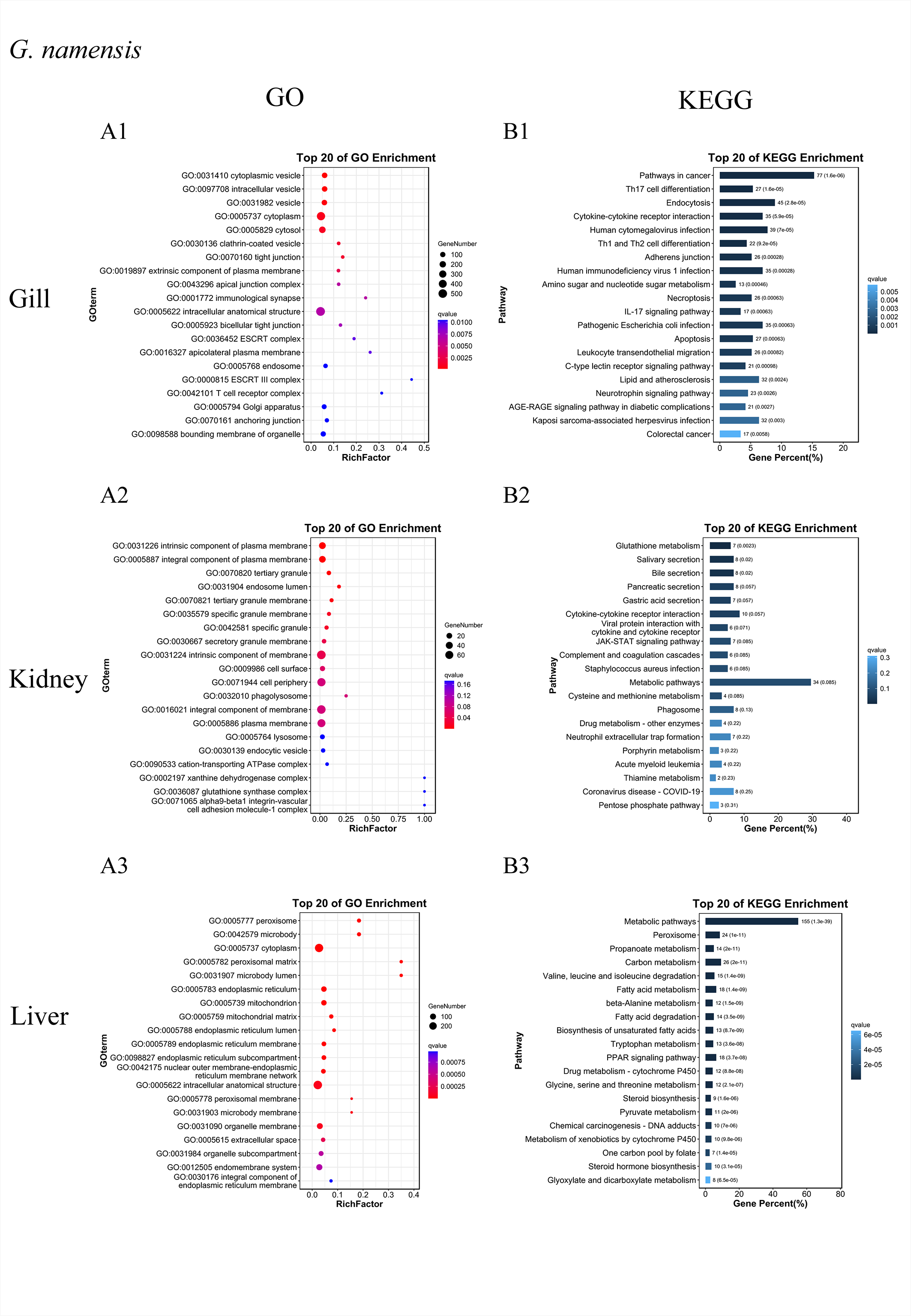

Supplement: Supplementary file 1 [file animals-15-00056-s001.zip › Suppl. Figures and Tables/Figures/Fig S3.tif]
